# The Nurse-Maid and the Mother of the Syphilitic Child

**Published:** 1878-11

**Authors:** James Nevins Hyde


### Original Lectures.

### THE NURSE-MAID AND THE MOTHER OF THE SYPHILITIC CHILD.

*(A Clinical Lecture Delivered at the Dermatological and Venereal Clinic, Rush Medical College.)*

BY JAMES NEVINS HYDE.

Reported by L. C. WATERS, Clinical Assistant.

I.

GENTLEMEN: You see this fresh-looking, blonde German girl, 14 years old. One week ago she came to me, accompanied by her mother, complaining of pain in her left side, and showed what looked like ulcers between several of her toes. They were really not ulcers, but, when superficially examined, might have been mistaken for such lesions. I examined her heart, and found that, although pulsating 120 times each minute, there was no valvular or other organic disease. She was probably somewhat alarmed by the fact of her presence in this place, and the fear of what might follow—this in part explained the acceleration of the pulse.

I will say here, that as soon as I looked at these interdigital sores, I recognized their character and import. Almost anyone, accustomed to careful inspection and mental classification of cutaneous lesions, could have done the same thing readily. But I want to call your attention to the ease with which we might have made a grievous blunder in this case. We might have said carelessly: "here is a young girl with sore toes and a pain in her left side—possibly neuralgic. She cannot give us any reason why she should suffer this way. We will therefore give her a salve for her ulcers, and a remedy for her neuralgia or cardialgia, and dismiss her for a week."

But we did not say nor do this. We pushed our investigation of the case till its origin and character were as clear as it was possible to be to us, whose facilities for such investigation are limited to this room. As she is now before us, we will first examine her physically, and then give her history.

I pass my hands along the back of her neck, and find here that the lymphatic glands, situated along the intermuscular septa, are moderately enlarged, but not tender. Two glands, one on either side of the nucha and just below the occiput—the sub-occipital glands—are as large as walnuts. This is a significant symptom. Looking into the throat, I find hyperæmia of the pharynx, and each tonsil enlarged to double its normal size. Upon the right pillar of the fauces is a superficial ulcer with purulent floor, as large as the section of a small split pea. The tint of her skin is delicately clear, yet, over the right arm, and in one or two places over the legs, are some small, irregularly dispersed, pin-head sized reddish papules, which have been scratched with the finger nails. We know this, because we find a little blood scale at the apex of each, and scratch lines in the vicinity. Exposing the genital organs, you see upon and about the vulva and the vulvo-crural integument, 10 to 12, irregularly oval, elevated and flattened patches, from 1 to 3 millimeters in height, and from 2 to 3 centimeters in diameter. One, situate upon the right labium minus, has so irritated the tissue beneath, that the lip is puffy and œdematous. All are now flesh-colored in hue, and though moist, destitute of much secretion. One week ago they were nearly as white as apple blossoms, and smeared over thickly with an offensive mucous secretion, of which I will remark, parenthetically, that it is in the highest degree capable of communicating the disease from which the patient is suffering. Six to eight more of these lesions surround the anus, similar in size, aspect and configuration. The inguinal glands of each side are enlarged and painless.

Let us stop to say a word about these vulvo-anal lesions. What name shall we give them and to what family do they belong? They have been variously called: (*Die breite Condylomata, die*

*feuchten oder nassenden Papeln, die flachen Condilome, papules humides, pustula fœda ani, pustules plattes, plâques muqueuses*), mucous patches, condylomata and mucous tubercles. They belong to the family of the papules, and are, in fact, giant papules with flattened apex and secreting surface—giant, because they form in regions where heat and moisture favor their extreme development—flattened, largely because they form in regions where they are subjected to friction and contact of apposed surfaces—secreting, because these regions are hot, moist and subjected to friction. Now look at the interdigital lesions upon the feet, which I said, a moment ago, could readily be mistaken for ulcers. Between the second and third, and between the fourth and fifth toes of this right foot, largely on the dorsal border of the quasi-mucous surfaces in apposition, you observe in each locality, not an ulcer, but an elevated lesion as large as a little finger nail, elevated as far as the skin of the lateral surface of the adjacent toe will permit, moist and secreting at the summit, but less moist and secreting less than the lesions about the vulva and anus. These also, during the last week, have lost some of their classical features—the improvement being due to the treatment pursued, of which I shall speak later. Similar, but less typical tubercles you see also between the toes of the left foot.

All the lesions I show you are identical in character. They are mucous patches, mucous tubercles, condylomata, or giant flattened papules. You may call them what you will, if only you understand the fact that they result from lawless or perverted cell development in parts where there is heat, moisture and friction, this lawlessness or perversion being due to the influence of the syphilitic virus, whose features in this case many of you have ere this probably recognized. The occurrence of these and allied lesions, is so constant and characteristic in the secondary stage of the disorder, that Zeissl proposed to call the latter the "condylomatous stage" of syphilis.

The recognition of this disease, gives us an explanation of the thoracic pain, of which the patient at first complained. Substernal and subcostal pains are frequent in the early periods after syphilitic infection. They are, in general, I believe, a nervous protest against the circulation and distribution of hydræmic and intoxicated blood.

Let us stop for a moment to enquire why we find upon the skin of this young girl, such an abundant development of mucous tubercles, while upon the skin of the next patient we chance to examine in the early period of development of constitutional syphilis, we may discover a symmetrical macular eruption over the face and chest, out of which may spring an equally distributed crop of papular or papulo-squamous syphilides. I think there is an explanation. This girl is of an age which entitles her to be classed with infantile patients. Infants and children have delicate skins, built up of rapidly growing cells, abundantly supplied with nutrient juices ; and their skins, under the influence of acquired syphilis, have a marked tendency to exhibit mucous lesions. The sex also is to be considered. Even without experience, reason would lead us not to look in these patients, for those classical symptoms which are displayed on the skin of the adult male, toughened by long exposure to friction during toil, at a period of life when cell growth is strictly limited to the demand for repair of daily waste.

Let us ask further how this child came to be affected in this way. She is but 14 years old, and has a remarkably innocent expression of countenance. It is difficult for us to believe that she has sinned sexually at her age, and her genitals bear no marks of indecent assault. I have questioned her in private, and can assure you that she is, in fact, as innocent as she is in appearance. I asked her if she had been at service as a nurse girl, and she answered that she had not. While making these inquiries, I carefully examined the epitrochlear glands of her arms—those found just above the inner condyle of the humerus, named by Chaussier, the epitrochlea—and found that that of the right side was enlarged, the left was unaffected. This certainly was significant. Further questioning elicited the fact that five months ago, she paid a two months' visit to an aunt in the country—her mother's sister. This woman, having lost a first husband, was united to a second husband two years ago, and was nursing a little three months old baby at the time of this visit to the country. The baby had been ill from the moment of its birth, and had a very sore mouth, "so sore that its mouth was kept constantly open." Our patient is positive that she never kissed this child—its mouth was so repulsive; nor had she ever slept with it.

The mother of our patient, who accompanies her, is evidently entirely in the dark as to the nature of her daughter's malady—as much so, indeed, as the daughter herself, who at first believed that she had "the whites." This woman is a widow, of healthy appearance, a mother of seven healthy children, who has never aborted. She denies the possibility of any venereal disease in her case, and is stricken with horror when informed as to the real character of her daughter's trouble. Then her memory is stimulated to the recollection of the fact that her son had told her that the father of the baby to whom we have reference, was affected with a "bad disease" at about the time of his marriage, and that his wife had been taking medicine for the same reason.

I think it is safe to assume that this infant had hereditary syphilis. It was of that age when we may look for manifestations of the disease. An infant suffering from mucous patches of the nasal and buccal cavities, is very apt to keep the mouth open, not because the mouth is sore, but because the nostrils are obstructed by the crusts which form there, and impede respiration. The offensive secretion from the mouth and nostrils of these little patients, is highly contagious. We will not now discuss the question whether this secretion is contagious for the parents of such a child; it is sufficient at present to know that for all others, unaffected with syphilis, this discharge is potent for mischief. It is true that this fact has been disputed by one author, but the proof against him is overwhelming. How can such children infect other individuals? I answer, by the act of kissing, by the mediation of spoons and utensils carried directly from the child's to another's mouth (tasting its pap, etc.), by the innocent contacts of sleep, and, as lately reported in one instance, by the aid of the finger of the infant, which was habitually sucked, and then thrust into the mouth of its nurse. We must not, of course, forget the contact of the child's mouth with the nurse's nipple, nor the possibility of conveying blood or pathological products from arm to arm in the process of vaccination. Professor Goodell, of Philadelphia, has recently stated that the liquor amnii of such infants at birth, is dangerous for the accoucheur.

By none of these methods was our patient infected. She is quite sure that she never kissed that offensive mouth. But, on further questioning, she admits that she carried this child, and often played with it, and, being pressed to state if she ever suffered from any wound at this time, she admits that she cut her hand upon a piece of glass. She points out to us the location of this wound, and lo, here we have revealed the site of her chancre, and the origin of all her subsequent trouble. You see upon the radial border of her right thumb, over the metacarpo-phalangeal articulation (that of the arm where we found the enlarged epitrochlear gland), a smooth elevated indurated nodule, oblong in shape, and as large as a pea, as resilient to the touch as if a smooth kernel of ivory were imbedded in its substance, and of an empurpled reddish hue, suggestive of blood-stasis and pigment. The wound inflicted by the glass, was doubtless in some way infected by contact with the secretion from the mouth of the infant she was then tending. Here was once then a chancre. But the wound has healed, and the chancre, if ever it ulcerated, has also healed, leaving us only this lesion—insignificant enough when carelessly viewed, but highly significant when carefully studied, to tell us by what portal the virus of the disease gained access to her lymphatic circulation.

Now, let me say of all these extra-genital chancres, that they are formidable only as they are liable to escape recognition. The syphilis which is dangerous, is the syphilis which has long been unrecognized. These extra-genital "initial scleroses," as the Germans call them (a good name, by the way), have two, and only two tolerably constant characteristics: first, induration; second, a vinous-red or purplish stain. Their configuration size and morphology, depend almost entirely upon the peculiarities of the pre-existing wound or abrasion, and we find such pre-existing wounds or abrasions, in well nigh every case. They may be irregular, scabbed, crusted or exulcerated sequelæ of typical vaccine vesicles; they may be curious alterations of simple fissures of the nipple or its areola; they may be mere dry scaly papules of the skin. I have recently treated three physicians in the vicinity of this city, for syphilis originating with chancres of the hand, produced by inoculation in gynecic or obstetric manipulations. At the time of my first examination, one had a deep, empurpled, indurated and thickened cicatrix of the finger; a second had a superficially ulcerated abrasion of the knuckle, resting upon a slightly indurated and elevated base; a third had a red and angry looking dense nodule, resulting from a "hang-nail."

Returning now to our patient, let us dwell for a moment on the question of the date of her infection. By the aid of her memory alone, we cannot fix this date. But we find that we have a latititude of from three to five months in which to allow for the two periods of incubation. For the period of incubation of the initial sclerosis, we may have from ten days to an entire month, and even more. For the period of secondary incubation, that which succeeds the appearance of the chancre, and extends to the date of the first appearance of constitutional symptoms, we may have from twelve days to three months, and even more. Loosely speaking, we may say that this generally requires about fifty days. Now I am quite sure that this child must have suffered from her disease for some time before her attention was directed to it. She was not expecting its advent, and it was not probably till it forced itself upon her attention, that she really concluded that she was ill. The time, therefore, between her visit to us and her primary accident, may be brought within the average usual in such cases.

Do you ask, why then was her initial sclerosis evident when she first exhibited constitutional disease? I answer that this is common. In nearly one half of all cases, especially of untreated cases, we can find the characteristic induration remaining when the first explosion of the general disease occurs. The more extensive the induration, the longer its survival. Often upon the genitals we find ulcerated chancres co-existing with syphilides of of the skin. The chancres have then survived their usefulness as nests of lawless cells. They are, indeed, no longer chancres —they have been transformed, altered in aspect and character, as Fournier has well shown. They have become secondary accidents—most often have been changed into elevated mucous patches or giant papules. There is then nothing anomalous in our discovery of this little nodule on the girl's finger at this time.

We thus conclude at the very point where the syphilitic history of our patient began; and, unless I am mistaken, all obscure histories are to be followed up in pretty much the same way. By the course pursued, we have done something else beside gaining instruction for ourselves. We have established by evidence that ought to be received in a court of law, first, the good moral character of this young girl, second, the guilt of the author of this chain of calamities, by which three presumably innocent individuals have been infected with a loathsome disease.

Lastly, we have been enabled, by recognizing the character of the malady, to put our patient on the road to a prompt recovery. I have already called your attention to the influence of a single week of treatment. We gave her mercury internally, and merely directed her to wash the affected parts carefully with soap and water, and afterwards to dust some dry calomel over the patches. This is a sufficiently simple treatment. In private practice, we should probably have been urged to pursue the most energetic measures possible, and then we might have subjected the patient to the fumes of mercury in a vapor bath. In the large cities we often send such cases to the bath houses, when we are sufficiently fortunate to find those the proprietors of which will not tamper with our patients. But this is rare enough. Those of you, however, who may practice in localities where these vapor bath establishments are not accessible, can accomplish the desired end, equally well though with more trouble, by the extemporaneous aid of a blanket, a kettle of hot water to supply vapor, a spirit lamp and a tin plate containing calomel or cinnabar, or the two in combination.

By these measures we can readily bring about the disappearance of the external leisons. Then should follow a continued, faithfully conducted, gentle course of mercurial medication, never pushed to the production of salivation or any other of the toxic effects of the metal, but pursued so that nutrition may be encouraged rather than impaired.

II.

The next patient before you is a woman of respectable appearance, 43 years of age, living with her husband, in the nineteenth year of her marriage. She tells us that in about one year and a half after her marriage her troubles began with an ulcerated sore throat, soon followed by what she describes as an attack of paralysis on the right side. In 1858, she had an attack of asthma, from which she entirely recovered. Since 1872 she has suffered from frequent attacks of what she calls "inflammatory rheumtism," affecting chiefly the extremities. For fifteen years she has suffered from ulceration affecting the right leg, and, now that she has removed from it the dressings, you can see what a deformed and useless member it is. During all these years a battle has been raging between ulceration and repair over almost its entire surface, and until very lately the destructive forces have had the better of the contest. At present you can see only eight or ten clean-cut, circular, granulating ulcers, in size varying from a silver quarter to a half of a dollar, distributed irregularly from below the external malleolus to the tubercle of the tibia. But, extending from each of these in various directions you can see well defined disks, with borders as clearly rounded as the lip of a saucer, made up of young, newly formed and imperfectly formed epidermis. These are the beds of old ulcers, let in, as it were, beneath the sound integument to the depth of one or two millimetres, just now flesh-colored in hue. If she continues to improve, these will eventually show as typical scars, at first pigmented, afterwards of a dead white color, unattached to the deeper tissues, capable of being pinched up between the finger and thumb, and showing by reflected light a certain degree of lustre.

Long disuse of this limb has brought about atrophy of the muscles, and a general feebleness of all the elements which make up its structure. There is no calf of the leg—it is of about the same circumference from below the knee to the ankle. At first the disuse and a dependent position, aided probably by bandages wrapped around the leg and not the foot, induced œdema of the foot—a puffiness, into which the finger could be pressed for some distance. But the years have changed all this. Hyperplastic exudation has transformed this foot into the swollen, dense and almost shapeless mass you now see before you—seamed with deep furrows, where once were the normal lines of depression, and bulging out here and there in masses, upon which my finger pressing with considerable force makes no impression. It is quite suggestive of elephantiasis, but it is a simple hypertrophy, due originally to the gravitation of the blood.

I now apply this eight-yard bandage snugly from the toes to the knee, covering in the ulcers and their dressings. This will aid in bringing about resorption of the plastic effusion in the foot, and in permitting the ulcers to heal by relieving them of blood pressure in excess from above. She finds the limb thus dressed far more comfortable.

(The patient was then removed from the room at the request of the lecturer.)

The woman who has just left us, gentlemen, is suffering from syphilis. She, however, suspects neither the cause nor character of her complaint, and I know of no good object to be gained by informing her of the facts. I have never asked her a question about the origin of her disease. It was Mr. Hutchinson, of London, I believe, who said that it was a useless cruelty to ask a a married woman if she had ever suffered from syphilis. Moreover, her statements, either upon one side or the other of such an enquiry would be without value to us. We know more upon this subject than she is in position to know. The mucous tract of the the vagina and vaginal envelope of the cervix is extensive; and primary syphilitic lesions here are often insignificant, short-lived and entirely unrecognized by women who are thus infected.

On the evidence furnished by this leg alone, we could affirm this woman to be syphilitic. But on this scrap of paper in my hand I have further interesting corroborative evidence taken from her own lips. It is a record of her pregnancies, and reads as follows:

1*st Pregnancy*—Boy and girl, twins; born at full term in 1861. Living at this date, and in good health.

2*d Pregnancy*—At the seventh and one-half month; miscarriage, in 1865.

3*d Pregnancy*—At full term, a boy; still-born, 1866.

*4th Pregnancy*—At full term, a boy; 1867. He lived to be eleven months old and died of "catarrh in the head."

*5th Pregnancy*—At full term, a girl; 1868. She died when nine months old; cause of death unknown.

*6th Pregnancy*—At full term, a boy; 1869. Now living.

*7th Pregnancy*—At full term, a girl; 1870. Died when nine months old; "teething."

*8th Pregnancy*—At full term, a girl; 1874. Died when four months old of "difficulty with the lungs."

*9th Pregnancy*—At full term, a girl; 1876. Died when three months old; "teething."

What a record! To him who can read it aright it tells a tale of marital infidelity, syphilis and the blighting effect of the parents' disease upon the fruit of their union. More, it tells, within a few years, of the date when the poison began to do its work. Let us examine it somewhat in detail.

It is clear that both parents were healthy when the first pregnancy was terminated. The twins then born are still living and healthy. About one year and a half after the marriage the mother had an ulcerated sore throat, the probable result of syphilis contracted from her husband after the birth of these children. Now, when both parents are syphilitic, and the syphilis of the wife is unrecognized and untreated, what is the general result so far as regards gestation? First, we have abortions from a blighted embryo. Then, as the years roll by (and, as you know, measuring by pregnancies means measuring by years), we have still-births; then a series of children born who survive but a few months. Lastly (as there is an end to almost everything in this world, even to the power to transmit syphilis to one's offspring, thanks to the conservatism of nature), we find children born who survive childhood but are yet apt to betray symptoms of the disease.

Now, look at the series in this instance occurring after the birth of the twins, and after the syphilitic infection we have demonstrated. First, a miscarriage; then a still birth at term; then a baby, who expired in its eleventh month, suffering from "catarrh in the head," as she called it—probably the snuffles from mucous patches in the nostrils;—then a child, who did not survive its ninth month; then came a boy, who is still living. At this point must have occurred one of those periods of repose which we constantly observe when syphilis is waning. The fury of the storm was over. But I have taken pains to examine this boy carefully—as he is not here, you will have to take my word for the truth of my statements—and his central upper incisor teeth are clearly pegged and notched, in the manner described by Hutchinson as typically characteristic of hereditary syphilis. After this boy were born three girls. All of them died of diseases to which we get no clue from the mother—they probably suffered from obscure visceral lesions due to infection. Look at the net result. One boy, the sole survivor of eight impregnated ova, after the first gestation with twins. How fatal should we consider an epidemic that could thus so nearly destroy an entire family!

The mother, who has just left us, is improving rapidly under the administration of the potassium iodide, of which she has gradually been enabled to take four grams daily. She is even enabled to get about on her feet for the first time in years. Had she been treated earlier, and her husband subjected to the same treatment, some of their children might have been saved. But she is approaching the period of the menopause, and the past is irrevocable. Locally we are dressing her ulcers with a weak mercurial ointment.

---

We have made no special investigations of the effect of quinia given in *diseased* conditions. In one case of syphilis which we observed, there was a marked increase in the red corpuscles 1,043,130 under the administration of cinchona sulphate.

There was in *health* a slight diminution in the number of red corpuscles and a marked increase in the number of the white, after the administration of a large dose of quinia sulphate. (Drs. Cutler and Bradford, *Amer. Jour. of the Med. Soci.*, Oct., 1878.)

